# Supplementary material for: Predicted coronavirus Nsp5 protease cleavage sites in the human proteome
Source: BMC Genom Data. 2022 Apr 4;23:25. doi: 10.1186/s12863-022-01044-y (PMC8977440; doi:10.1186/s12863-022-01044-y)
Supplement: Supplementary file 5 — Additional file 5: Figure S1. Interaction scores vs max NetCorona score. [file 12863_2022_1044_MOESM5_ESM.pdf]

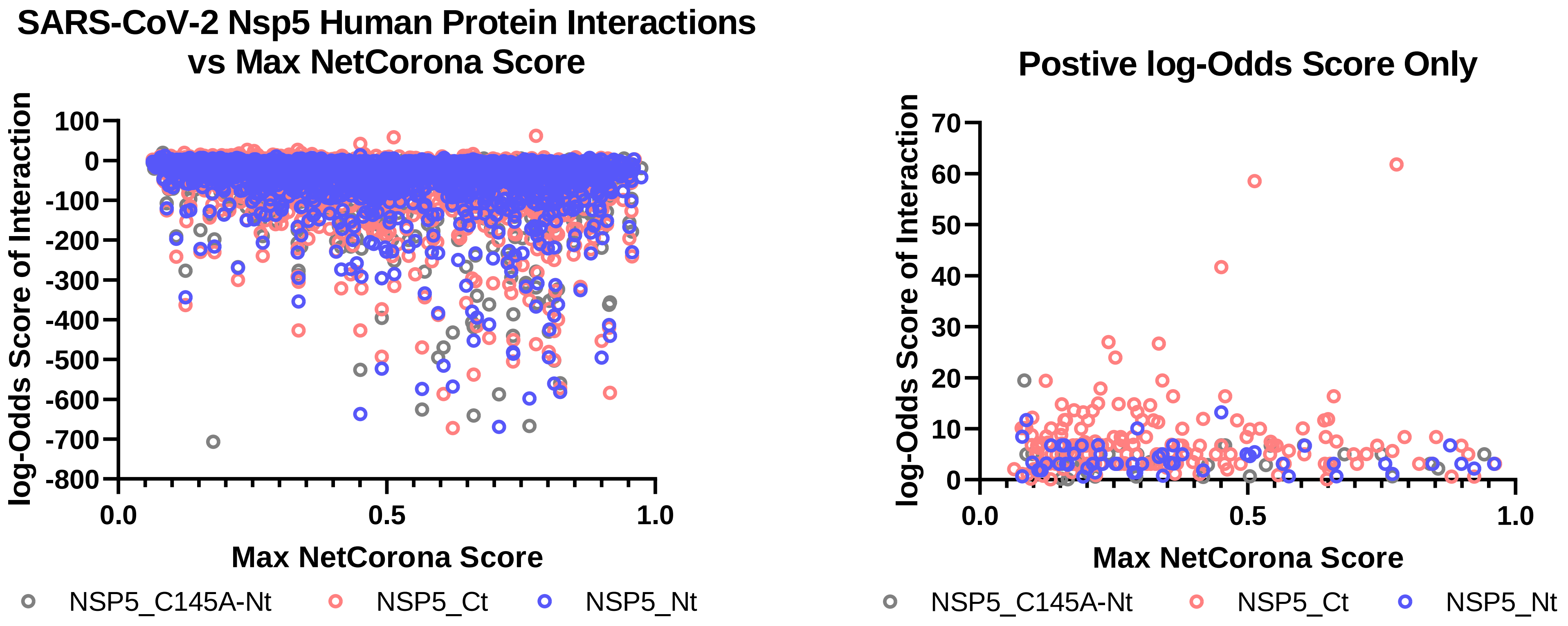

	Max NetCorona Score vs. NSP5_C145A-Nt	Max NetCorona Score vs. NSP5_Ct	Max NetCorona Score vs. NSP5_Nt
Pearson r			
r	-0.1762	-0.2902	-0.1966
95% confidence interval	-0.2278 to -0.1235	-0.3419 to -0.2367	-0.2417 to -0.1506
R squared	0.03103	0.08422	0.03866
P value			
P (two-tailed)	<0.0001	<0.0001	<0.0001
P value summary	****	****	****
Significant? (alpha = 0.05)	Yes	Yes	Yes
Number of XY Pairs	1325	1165	1712

Additional File 5: Figure S1 Nsp5-human protein interaction data from Samavarchi-Tehrani *et al.* (2020), plotted against the maximum NetCorona score for human proteins from the "One Protein Per Gene" dataset. log-Odds Score of Interaction obtained from Samavarchi-Tehrani P, Abdouni H, Knight JDR, Astori A, Samson R, Lin Z-Y, Kim D-K, Knapp JJ, St-Germain J, Go CD et al: A SARS-CoV-2 – host proximity interactome. bioRxiv 2020:2020.2009.2003.282103. Also available from covid19interactome.org
