# Supplementary material for: Predicted coronavirus Nsp5 protease cleavage sites in the human proteome
Source: BMC Genom Data. 2022 Apr 4;23:25. doi: 10.1186/s12863-022-01044-y (PMC8977440; doi:10.1186/s12863-022-01044-y)
Supplement: Supplementary file 7 — Additional file 7: Figure S2. ASA vs NetCorona score. [file 12863_2022_1044_MOESM7_ESM.pdf]

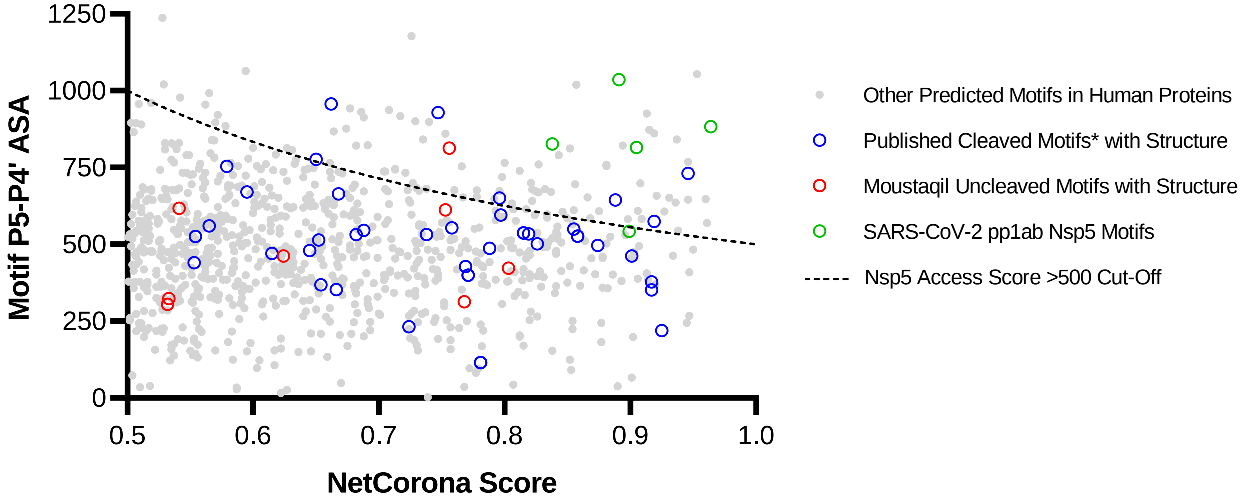

Additional File 7: Figure S2 Accessible surface area (ASA) of predicted and known Nsp5 motifs plotted against NetCorona scores, with data published by others highlighted. The Nsp5 access score cut-off of >500 is displayed for reference. *Published cleaved motifs** indicates that a specific peptide motif with a glutamine at P1 in a human protein has been shown to be cleaved by coronavirus Nsp5, by using an *in vitro* cleavage assay and running the product on an SDS-PAGE, or by predicted using N-terminomics. These motifs were published in the following papers:

Koudelka T, et al. N-Terminomics for the Identification of In Vitro Substrates and Cleavage Site Specificity of the SARS-CoV-2 Main Protease. Proteomics. 2021;21(2):e2000246

Moustaqil M, et al. SARS-CoV-2 proteases PLpro and 3CLpro cleave IRF3 and critical modulators of inflammatory pathways (NLRP12 and TAB1): implications for disease presentation across species. Emerg Microbes Infect. 2021;10(1):178-95.

Pablos I, Machado Y, de Jesus HCR, Mohamud Y, Kappelhoff R, Lindskog C, et al. Mechanistic insights into COVID-19 by global analysis of the SARS-CoV-2 3CL(pro) substrate degradome. Cell Rep. 2021;37(4):109892.

Yucel N, Marchiano S, Tchelepi E, Paterlini G, McAfee Q, Nimmagadda N, et al. Prediction and validation of host cleavage targets of SARS-CoV-2 3C-like protease. bioRxiv. 2022:2022.01.17.476677.
